# Effects of 5-aza-2´-deoxycytidine on primary human chondrocytes from osteoarthritic patients

**DOI:** 10.1371/journal.pone.0234641

**Published:** 2020-06-23

**Authors:** Shirin Kadler, Özlem Vural, Jennifer Rosowski, Luzia Reiners-Schramm, Roland Lauster, Mark Rosowski

**Affiliations:** Department of Medical Biotechnology, Institute of Biotechnology, Technische Universität Berlin, Berlin, Germany; Università degli Studi della Campania, ITALY

## Abstract

Chondrocytes, comparable to many cells from the connective tissue, dedifferentiate and end up in a similar fibroblastoid cell type, marked by the loss of the specific expression pattern. Here, chondrocytes isolated from osteoarthritic (OA) patients were investigated. The OA chondrocytes used in this work were not affected by the loss of specific gene expression upon cell culture. The mRNA levels of known cartilage markers, such as SOX5, SOX6, SOX9, aggrecan and proteoglycan 4, remained unchanged. Since chondrocytes from OA and healthy tissue show different DNA methylation patterns, the underlying mechanisms of cartilage marker maintenance were investigated with a focus on the epigenetic modification by DNA methylation. The treatment of dedifferentiated chondrocytes with the DNA methyltransferase inhibitor 5-aza-2´-deoxycytidine (5-aza-dC) displayed no considerable impact on the maintenance of marker gene expression observed in the dedifferentiated state, while the chondrogenic differentiation capacity was compromised. On the other hand, the pre-cultivation with 5-aza-dC improved the osteogenesis and adipogenesis of OA chondrocytes. Contradictory to these effects, the DNA methylation levels were not reduced after treatment for four weeks with 1 μM 5-aza-dC. In conclusion, 5-aza-dC affects the differentiation capacity of OA chondrocytes, while the global DNA methylation level remains stable. Furthermore, dedifferentiated chondrocytes isolated from late-stage OA patients represent a reliable cell source for *in vitro* studies and disease models without the need for additional alterations.

## Introduction

The loss of environmental signals occurring during injuries in multicellular organisms leads to a stepwise reprogramming or dedifferentiation of cells. On the other hand, cell fate is stabilized by epigenetic modifications, such as those found in histone tail marks or DNA methylations. In this context, tissue-dependent differentially methylated regions can be identified in differentiated cells. These cell type-specific sequences are demethylated in the tissue regarded and are associated with active histone marks, while these sequences in other tissues are transcriptionally inactivated by methylation [1]. Therefore, the methylation status of key regulatory sequences represents a crucial factor in differentiation processes and cell phenotype maintenance and should be taken into consideration for successful regenerative applications [2,3]. The enzymes responsible for the DNA methylation are encoded by the genes DNMT1, DNMT3a and DNMT3b (DNA methyltransferases: DNMTs). However, the introduction and the removal of DNA modifications are necessary for cellular development. No *in vivo* mechanism of direct DNA demethylation has been described so far, nevertheless, modified intermediates, such as 5-hydroxymethylcytosine, 5-formylcytosine and 5-carboxylcytosine, have been found [4]. These further modifications of 5-methylcytosine are catalyzed by the TET (ten-eleven translocation) family.

Cells dedifferentiate upon *in vitro* cell culture, caused by the withdrawal of tissue-specific microenvironmental cues, and this reflects the loss of tissue specificity. The dedifferentiation process of isolated human chondrocytes affects the epigenetic pattern of the cells on a DNA methylation and histone modification level [5,6]. Alterations in DNA methylation are also identified in comparative studies of healthy chondrocytes with cells from osteoarthritic tissues [7,8].

Osteoarthritis (OA) is the most common disease of the osteochondral unit. It is characterized by the loss of permanent cartilage, reduced joint spaces, osteophyte development, subchondral bone cyst formation and sclerosis. Clusters of proliferating chondrocytes emerge in early osteoarthritic cartilage to compensate for the loss of matrix integrity. The proliferation of hitherto resting articular chondrocytes is initiated by changes in the environment of the pericellular matrix. The disruption of the connection between chondrocytes and their pericellular matrices compromise the HA-CD44 signaling (hyaluronan and its receptor) and results in the upregulation of matrix metalloproteinases with a decreased survival of cells [9]. Fissures in the cartilage extracellular matrix can be detected with the progression of the disease. The degradation leads to an increase in oxygen tension that further accelerates the process of tissue destruction. The subchondral bone layer is subsequently affected, marked by tissue mass reduction. Furthermore, the subjacent calcified layer expands into the articular zone, and tissue vascularization is induced in late OA stages. The disease does not only affect the osteochondral unit of the joint but also the ligaments and the synovium [10]. Pharmaceutical intervention is the only option for patients to reduce pain since OA is incurable. The replacement of the joint by surgery in late state OA is the only option for patients.

Tissue engineering applications provide a promising strategy to regenerate the damaged tissues. In these technologies, the dedifferentiation process is reversed by the cultivation of dedifferentiated cells or stem cells in appropriate conditions capable of inducing the differentiation desired [11]. The cultivation conditions are oriented towards the *in vivo* process of chondrocyte differentiation and cartilage development by endochondral ossification [12]. The process of endochondral ossification is initiated by the condensation of mesenchymal stem cells following differentiation steps of proliferative chondrocytes, hypertrophic chondrocytes and subsequent bone formation [13]. In parallel, articular cartilage differentiation and joint formation are regulated in a precisely orchestrated developmental process [14]. Sets of marker molecules are well-described for both the differentiation of articular chondrocytes and the differentiated cartilage tissue. These sets include transcription factors, such as SOX5 and SOX9, signaling molecules from the FGF-, BMP- and WNT-family and matrix molecules, such as collagen type 2 and aggrecan [13,15].

The molecules 5-azacytosine (5-aza) and 5-aza-2´-deoxycytosine (5-aza-dC) were first synthesized in 1964 [16] and have had FDA approval for myelodysplastic syndrome treatment since 2004 and 2006, respectively. This base is a modification of cytosine, replacing the carbon at position 5 by nitrogen. This small alteration inhibits the functionality of DNMTs by forming an irreversible covalent bond between the DNMT and the 5-aza-dC-incorporated DNA strand [17]. Hence, the incorporation of 5-aza-dC into the DNA leads to a decreasing amount of functional DNMTs in the nucleus. It is reported by Cameron and colleagues [18] and many others that cell lines treated with 5-aza-dC led to a decrease in DNA methylation and expression of formerly repressed genes.

In order to assess the impact on regenerative applications, we analyzed the influence of a DNMT inhibitor on chondrocytes isolated from patients with late-stage OA. The differentiation potential towards adipogenic, osteogenic and chondrogenic lineages was tested, and DNA methylation levels for specific target sites were analyzed.

## Material and methods

### Ethics statement

The acquisition of cartilage tissue was approved by the Ethics Committee Charité Berlin (EA1/047/09) and all patients gave written consent for this research.

### Cell culture

Cartilage was extracted from hip and knee replacement surgeries of OA patients. Samples were isolated from non-weight bearing sections with no visible lesions. Human chondrocytes are the sole cellular component in articular cartilage, therefore, no sorting strategy was necessary. The extracted cartilage was washed twice with phosphate-buffered saline (PBS) and briefly with 80% (v/v) ethanol, followed by PBS and dissected from the underlying bone. The tissue was cut in small 2 x 2 mm pieces using a scalpel.

The pieces were digested with 1 mg/ml protease K (Sigma Aldrich) for 30 min, washed in PBS and digested in collagenase (2 mg/ml) overnight. A 70 μM cell strainer was used to remove the extracellular matrix debris from the cells. Chondrocytes were counted, and 5 x 10^5^ cells were lysed for nucleic acid isolation. All the remaining cells were seeded in a T25 cell culture flask for adherent growth using DMEM with 10% FBS and 1% penicillin/streptomycin. Chondrocytes were passaged at 80% confluence. Cells were used at passage 4 to passage 13 for the experiments. No indications of senescence were found, even at late passages.

### AZA treatment

For the differentiation studies, the non-confluent adherent cells were treated with 1 μM 5-aza-dC (100 mM stock solution in 25% acetic acid) in growth medium for a minimum of three to four weeks to ensure the incorporation of 5-aza-dC into the DNA. Fresh 5-aza-dC was added every 24 h, while the complete medium exchange was performed every other day. Afterwards, the chondrocytes were detached and used for osteogenic, adipogenic and chondrogenic differentiation studies or lysed for specific gene expression analysis on mRNA level.

### Osteogenic differentiation

Cells were seeded in 6-well plates (2.5 x 10^5^/well), 5-aza-dC was removed, and media was changed to osteogenic differentiation media containing 10 mM beta-glycerophosphate, 10 nM cholecalciferol (vitamin D3), 100 μM ascorbate phosphate and 10 mM dexamethasone in final concentrations for 28 days. The medium was changed every other day. Differentiation was visualized by Alizarin Red staining and confirmed with quantitative PCR (qPCR) by testing the mRNA level for osteopontin (OPN).

### Alizarin Red staining

The medium was carefully aspirated from the cell culture wells and washed twice with dH_2_O. Alizarin Red solution (2% in dH_2_O at pH 4.1–4.3) was applied to the wells and incubated at room temperature for 5–10 min. The staining solution was removed carefully, and the wells were gently washed twice with dH_2_O. Pictures were taken immediately.

### Adipogenic differentiation

The cells were seeded in 6-well plates (2.5 x 10^5^/well), 5-aza-dC was removed, and media was changed to Adipogenic differentiation media containing 10 μg/ml insulin, 500 μM 3-isobutyl-1-methylxanthine, 0.2 mM indomethacin and 1 μM dexamethasone. The medium was changed every other day. After 28 days, differentiation was visualized by Oil Red O staining and confirmed with qPCR by testing the mRNA level for fatty acid binding protein 4 (FABP4).

### Oil Red O staining

The media was removed, and the cells were gently washed with PBS to visualize the lipid vesicles in them. The cells were fixed in 10% formalin for 10–30 min. The formalin was removed, and the wells were washed using, firstly, PBS and then 60% isopropanol. Subsequently, the wells were emptied to dry completely. Three parts of the Oil Red O stock solution (3% in isopropanol) were mixed with two parts of dH_2_O before staining to prepare the staining solution. After 10 min, the working solution was filtered and added to the dried wells for 10 min. The staining solution was removed, and the cells were gently washed four times with dH_2_O. The cells were prevented from drying out. Staining was examined under the microscope and pictures were taken.

### Chondrogenic differentiation

Cells were seeded in a 24-well ultra-low attachment plate (10^6^ cells/well, Corning) in DMEM supplemented with 10% FBS and 1% penicillin/streptomycin without 5-aza-dC. During this cultivation step, the cells undergo mesenchymal condensation, forming one self-organized aggregate. After condensation is completed (one to two weeks), the media was changed to chondrogenic conditions using serum-free DMEM (1% penicillin/streptomycin) with 100 nM dexamethasone, 200 nM ascorbate phosphate, 40 μg/ml L-proline, 100 μg/ml sodium pyruvate, 1% ITS-Premix and 10 ng/ml TGF-beta-3 (PromoKine) in final concentrations for four weeks. The media was changed three times a week. Differentiation was visualized after cryosectioning by proteoglycan staining with Alcian blue and Safranin O/Fast green on glass slides. The expression of collagen type II chain α1 (COL2A1) and aggrecan (ACAN) was further confirmed by qPCR and the glycosaminoglycan content was determined.

### Cryosections

Cell condensates were embedded in O.C.T Compound (Tissue-Tek), snap frozen and stored at -80°C. The tissue was cut at -16 to -18°C using a specialized knife for hard tissues and 8 μm sections were placed on glass slides (Histobond, Marienfeld, Germany). After drying, slides were stored at -20°C. Samples were cut at a thickness of 25–30 μm and were collected in 2 ml centrifuge tubes for RNA extraction. A quantity of 1 ml QIAzol (Qiagen, Germany) was added, and RNA samples were stored at -80°C until extraction.

### Immunostaining

Slides were thawed at room temperature for 10 min and then fixed in ice cold acetone for 20 min. After 3 washing steps with PBS, the sample was blocked with 10% serum for 30–60 min to prevent unspecific binding of antibodies. The incubation of the primary antibody (collagen type II, Merck Millipore #MAB8887, 1:20) was done overnight at 4°C. Then 3 washing steps with PBS were performed prior to the addition of the secondary antibody (goat anti mouse Alexa594, Invitrogen #A11005, 1:200) for 45 min at room temperature. During the last 10 min of this step Hoechst staining was performed, adding the dye solution at a final concentration of 0,5 μg/ml to the sample. After a last washing step, the sample was protected with a coverslip using an aqueous cover solution. An isotype control or a no primary antibody control was prepared simultaneously to detect background intensity. Pictures were taken on the same day.

### Alcian blue staining

Sample slides were thawed at room temperature for 30 min and then fixed in 10% formalin for 10 min. After fixation has been completed, the slides were brought to dH2O and then incubated in 3% acetic acid for 3 min. Alcian blue staining, using 1% Alcian blue 8G (Sigma Aldrich) in 3% acetic acid at pH 1.5–2.5 for 15–20 min, followed. The slides were washed in water, then dehydrated and mounted. Glycosaminoglycans were stained in turquoise to light blue.

### Safranin O/Fast green staining

Sample slides were thawed at room temperature for 30 min and then fixed in 10% formalin for 10 min. The slides were stained for 10 min in freshly mixed Weigert’s iron hematoxylin solution (Roth). In order to differentiate the color, the slides were washed in running tap water for 10 min. Nuclear counterstain was performed by adding the slides to Fast green solution (0.05% in dH_2_O) for 5 min. Sections were briefly (10 s) rinsed with 1% acetic acid and then stained with Safranin O (0.1% in dH_2_O) for 5 min. The slides were dehydrated and mounted. Glycosaminoglycans were stained in red, nuclei in black and cytoplasm in light green.

### Measurement of glycosaminoglycans

The content of glycosaminoglycans was measured using DMMB staining (1,9 dimethyl methylene blue). Therefore, the condensates were firstly digested overnight at 56°C in 700 μl digestion buffer (50 mM Tris-HCl, 10 mM Na Cl, 3 mM MgCl2, 1% Triton X 100 at pH 7.9) containing proteinase K (50 ng/ml). The digestion was stopped at 90°C for 20 min. The sample was divided in half. One volume of 350 μl was used to isolate genomic DNA (Macherey Nagel, NucleoSpin Tissue Kit) and the other half was further processed by digesting DNA with three units of DNase I at 37°C overnight. The sample was centrifuged at 13,000 x *g* for 20 min, and the supernatant was divided into three samples, each of 100 μl. A quantity of 1 ml of DMBB solution (0.0016% DMBB, 5% EtOH, 0.2 M GuHCl, 0.2% sodium formate and 0.2% formic acid, adjusted to pH 1.5 with HCl) was added to each 100-μl sample and incubated for 30 min on a shaker. The color complex was pelleted by centrifuging at 12,000 x *g* for 10 min. The supernatant was removed, and the pellet was resuspended on a shaker for 30 min by adding 300 μl DMBB de-complexation solution (4 M GuHCL in 50 mM sodium acetate (pH 6.8) + 10% 1-propanol). The OD at 656 nm was measured in triplicate. A standard series of chondroitin sulfate in DMBB de-complexation solution (0–60 μg/ml) was performed for quantification. Column-isolated DNA was quantified spectrometrically with a NanoDrop2000 (Thermo Scientific, USA) and glycosaminoglycan values were presented as a ratio normalized to the DNA content of related samples.

### RNA isolation

Adherent cells were lysed directly on the plate or after collection. The total RNA was extracted and purified using a Nucleospin RNA II Kit (Macherey-Nagel, Germany), according to the manufacturer’s protocol. Samples were snap frozen and cut into 25–30 μm sections before lysis for RNA isolation from cell condensates. The sections were transferred to 1 ml QIAzol (Qiagen) and stored at -80°C until isolation. Phenol/chloroform extraction of total RNA was performed. The RNA pellets were resuspended in 20–60 μl RNase free water, depending on the pellet size. The RNA content was measured spectrometrically at 260 nm using a NanoDrop2000 (Thermo Scientific, USA).

### Genomic DNA isolation

Genomic DNA was extracted with the NucleoSpin Tissue Kit (Macharey Nagel), according to the manufacturer’s protocol. The DNA was eluted in 60 μl dH_2_O measured spectroscopically and stored at -20°C.

### Bisulfite sequencing

An amount of 500 ng of genomic DNA was bisulfite-converted using an EZ DNA Methylation Gold Kit (Zymo Research Europe, Germany). The sequence of the proximal promoter of PECAM was obtained from the UCSC genome browser (human genome assembly hg18). All cytosine bases not in a CpG dinucleotide context were converted to thymine in a text editor, and PCR primer was designed on the converted template using the primer3 software [19,20]. The PCR product spanned the promoter from -164 bp to +285 bp regarding the transcriptional start.

### Proliferation assay

The cells were harvested by trypsinization, washed twice with PBS and prepared in a cell density of 2 x 10^6^ cells/ml. A 2X CFDA-SE (10 μM) working solution was prepared. Equal volumes of cell suspension and the 2X working solution were mixed and incubated for 7 min at room temperature away from light. The labeling was stopped by adding five volumes of cold growth medium and incubation of 5 min on ice in the dark followed. The cells were centrifuged and washed twice with growth medium and then seeded in the desired density to perform the assay. Cells were seeded at a high density to exclude cell proliferation by contact inhibition for the nonproliferating control. Other wells were seeded at 20% confluence to monitor cell doublings and were treated with 0.5 to 5 μM 5-aza-dC (Sigma Aldrich).

### Karyotyping

Dedifferentiated chondrocytes from three different donors were cultivated for 4 weeks with 1 μM 5-aza-dC prior to karyotype analysis. The karyotyping was performed at the Institute of Human Genetics at Charité Berlin.

### cDNA synthesis and quantitative PCR

An amount of 200 ng total RNA was reverse transcribed using the TaqMan kit (Thermofisher). The cDNA level was analyzed on an MxPro3005 (Agilent Technologies) using a SensiFast SYBR No-ROX Kit (Bioline, UK), 1 μl cDNA and 250 nM of each primer. The qPCR was done using the following thermal profile: 95°C for 2 min, then 40 cycles of 12 s at 95°C, 7 s at 64°C and 3 s at 72°C with additional melt point analysis. Gene expression was calculated using an amplification efficiency (E) of 1.95 and values were normalized on reference gene UBE2D2 (NM_181838) expression. The sequences of the qPCR primers and the gene identifiers used are listed in the S1 Table. The students t-test was performed for statistical analysis and differences in expression for p-values < 0.05 = * (< 0.01 = **, < 0.001 = ***, < 0.0001 = ****) were regarded as significant. Paired or unpaired t-tests were performed, depending on the experimental setup, using GraphPad Prism version 6 for Mac (GraphPad Software, La Jolla, California, USA).

## Results

**Fig 1:**
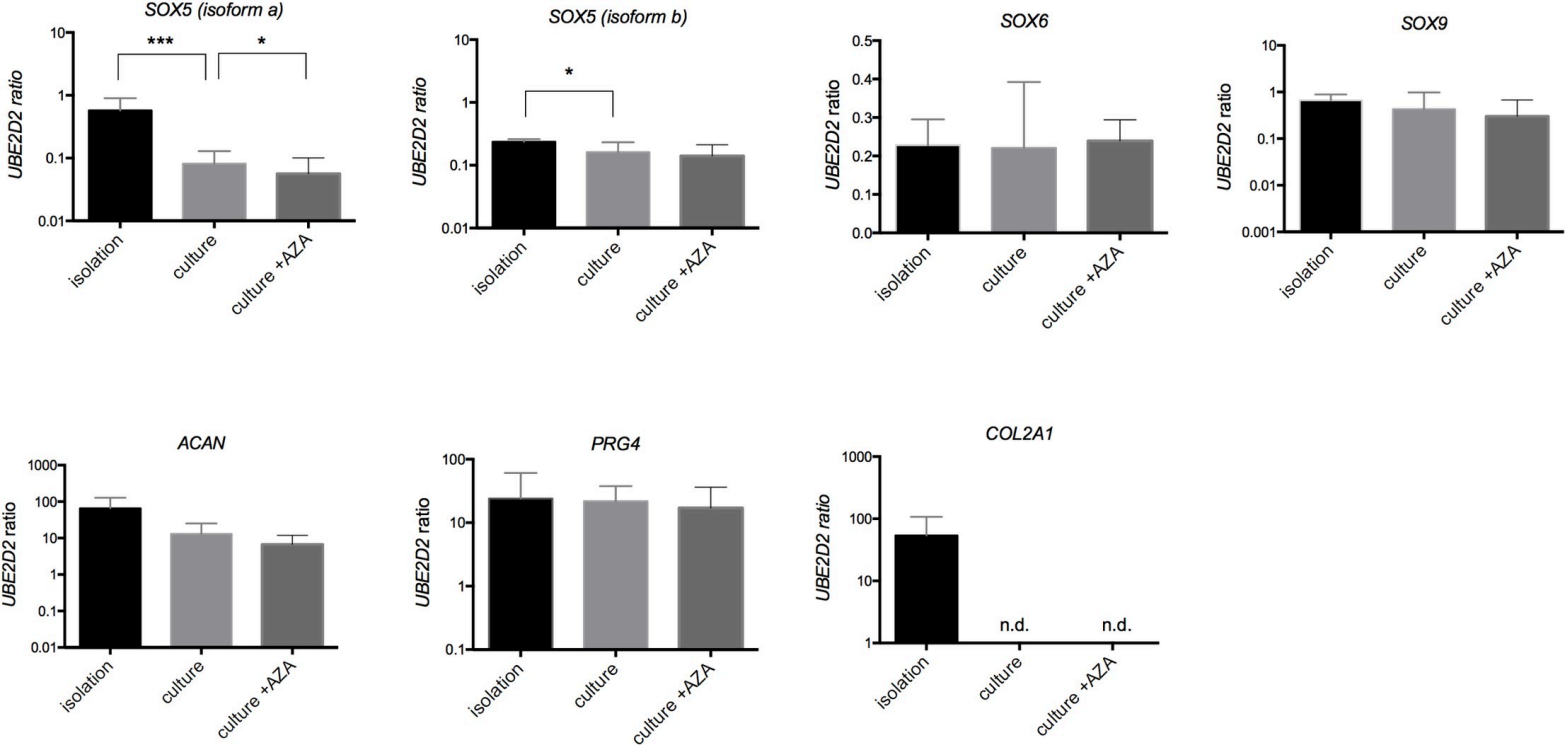
Gene expression in osteoarthritic (OA) chondrocytes w/o 5-aza-2´-deoxycytosine (5-aza-dC) treatment. The mRNA levels of *SOX5*, *SOX6*, *SOX9*, *ACAN*, *PRG4* and *COL2A1* normalized on reference gene expression *UBE2D2*. Cells were cultivated for four passages and then treated with 1 μM 5-aza-dC for four weeks. Statistical analysis was done using a ratio paired t-test. Values are mean ± SD (n = 10; *p < 0.05, **p < 0.01, ***p < 0.001).

Primary chondrocytes dedifferentiate upon isolation from articular cartilage characterized by a morphological transition from round to fibroblastoid shape. Interestingly, the impact on marker molecule expression seems to be bivalent (Fig 1). Although both isoforms of transcription factor SOX5 tested were downregulated and the extracellular matrix molecule encoded by COL2A1 was not even detectable after cultivation, the mRNA levels for the cartilage-specific markers SOX6, SOX9, ACAN and proteoglycan 4 were unaffected after several weeks of cultivation.

The impact of the DNA methylation on the marker gene expression was assessed by treating cultivated chondrocytes with the DNMT inhibitor 5-aza-dC. The treatment with 1 μM 5-aza-dC for four weeks did not alter the expression of SOX5L (isoform b), SOX6, SOX9, PRG4 or ACAN. Only SOX5M (isoform a) was further downregulated after 5-aza-dC administration. COL2A1 was not re-expressed after the treatment with the DNMT inhibitor (Fig 1).

**Fig 2:**
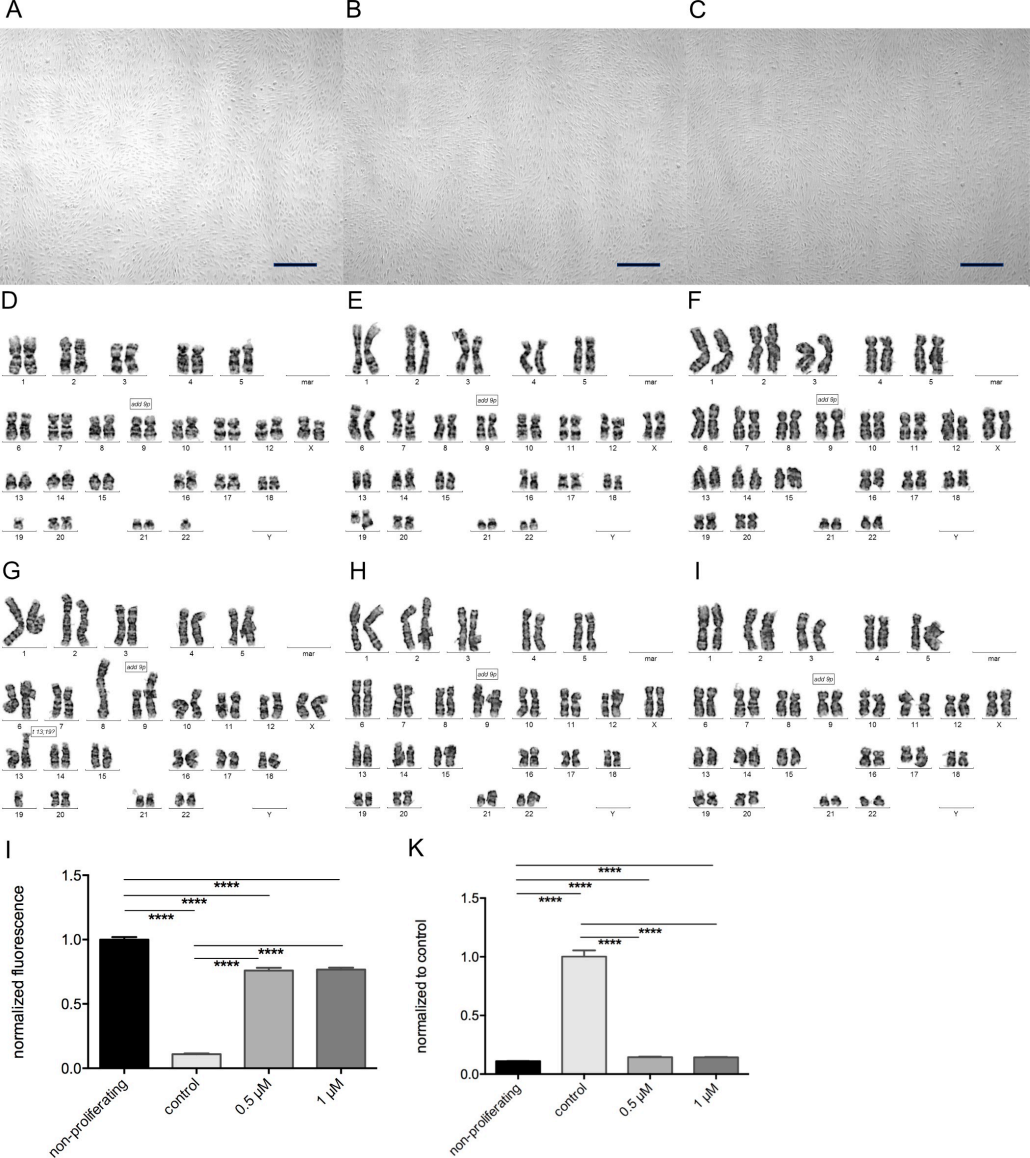
Effect of 5-aza-dC on chondrocyte morphology, stability and proliferation. (A-D) Dedifferentiated chondrocytes were cultured in the presence of 10 μM (B) and 20 μM (C) 5-aza-dC or without (A) to observe morphological changes in these cultures. (D-I) Dedifferentiated chondrocytes from three different donors were treated with 1 μM 5-aza-dC (D-F) for 4 weeks and karyotyping was performed to exhibit chromosomal aberrations compared to controls (G-I). (J,K) Carboxyfluorescein succinimidyl ester-labeled chondrocytes were treated with 0.5 and 1 μM for 16 days. The values represent mean fluorescence (normalized on nonproliferative control (J) and to untreated chondrocytes (K)). Statistical analysis was done using a ratio paired t-test. Values are the mean ± SD (n = 10; *p < 0.05, **p < 0.01, ***p < 0.001). Measure bars (A-C) represents 500 μm.

Since the incorporation of 5-aza-dC could lead to chromosomal instabilities, we analyzed the karyotype. In Fig 2D–2F, our results show no increased incidence of chromosomal aberrations compared to untreated chondrocytes (Fig 2G–2I). Microscopically, neither the cells alter their appearances, nor did we find an accumulation of detached cells even with increasing amount of 5-aza-dC (Fig 2A–2C).

Articular chondrocytes do not proliferate in their natural environment, but the cells reenter the cell cycle after isolation and *in vitro* cultivation. Since the incorporation of 5-aza-dC during proliferation is necessary to inhibit the DNMTs, we performed a proliferation assay. The half-life period of 5-aza-dC varies between 4 and 12 h [17,21], therefore, a concentration of 1 μM as well as 0.5 μM were used during this assay. Significant proliferation inhibition in chondrocytes was observed in our experiments with as little as 0.5 μM 5-aza-dC in the growth medium (Fig 2J and 2K).

**Fig 3:**
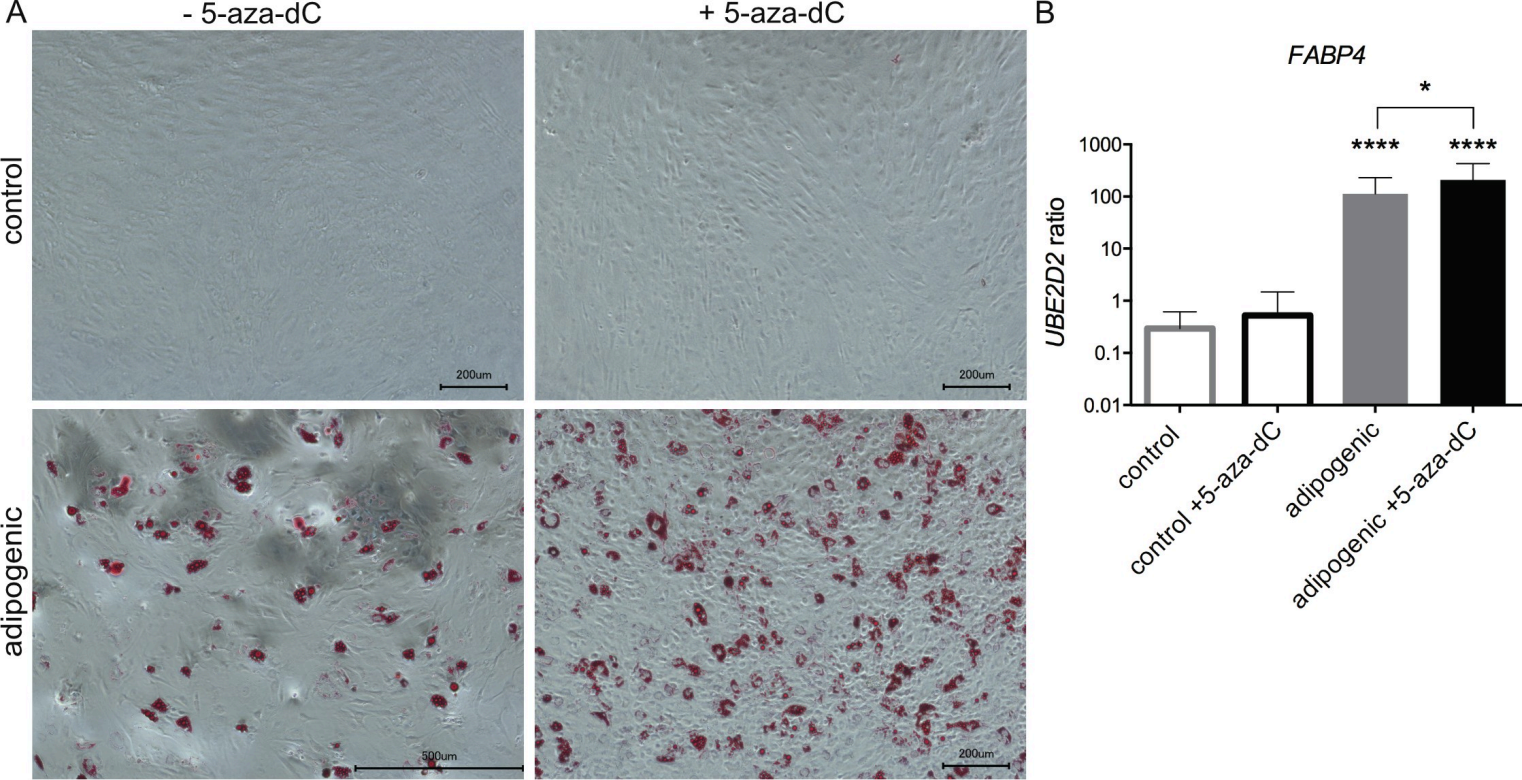
Influence of 5-aza-dC on the adipogenic potential of OA chondrocytes. After 5-aza-dC treatment, OA chondrocytes were differentiated towards adipocytes. (A) After four weeks, lipid vesicles were stained with Oil Red O. Pictures were taken at the same magnification. (B) Gene expression of *FABP4*. Expression was normalized on reference gene *UBE2D2*. Values are mean ± SD and statistical analysis was done using a paired ratio t-test. (n ≥ 4; *p < 0.05, ****p < 0.0001).

**Fig 4:**
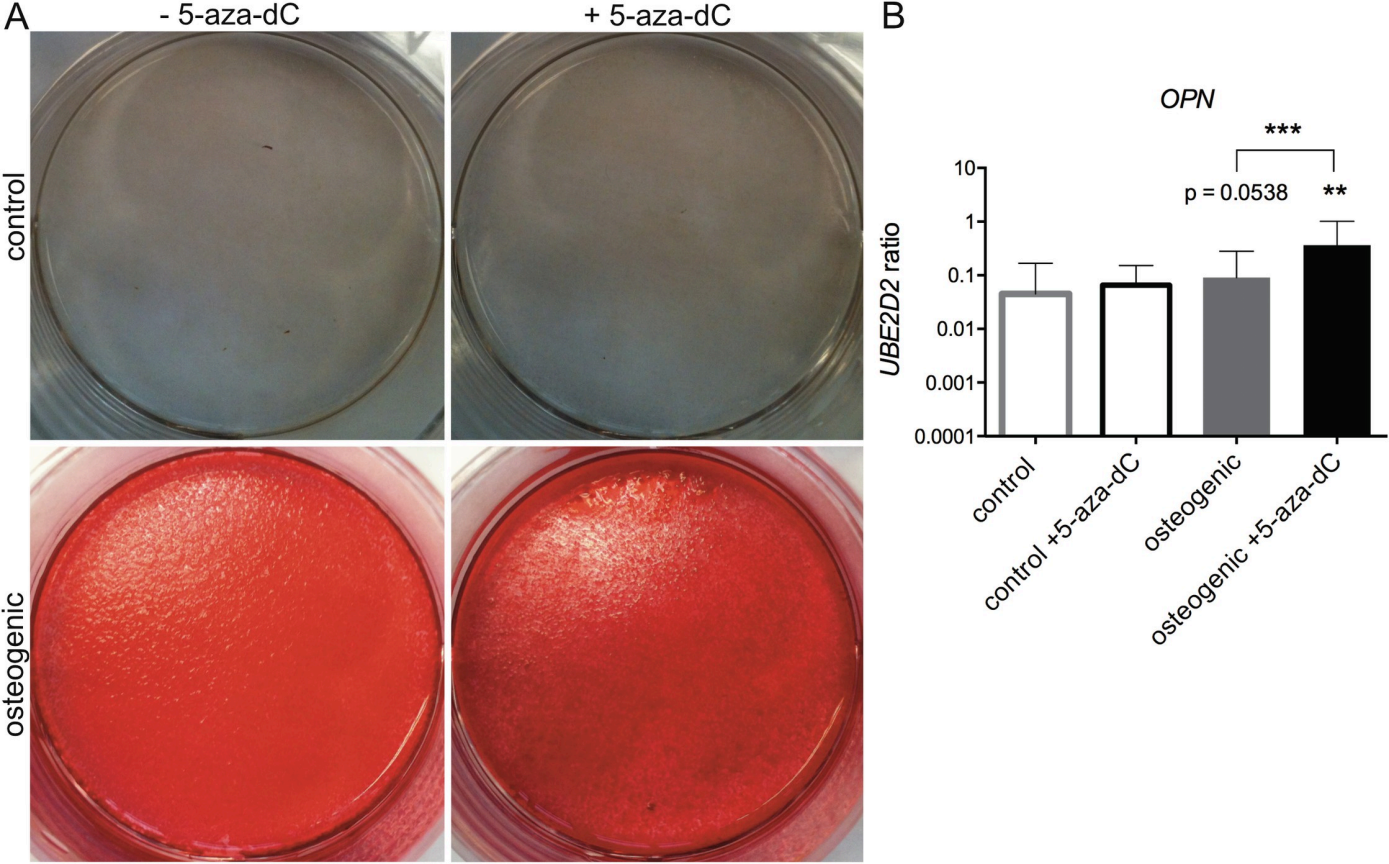
Influence of 5-aza-dC on the osteogenic potential of OA chondrocytes. After 5-aza-dC treatment, the OA chondrocytes were differentiated towards osteoblast. (A) After four weeks, the calcified matrix was stained with Alizarin Red. (B) Gene expression of *OPN* was measured and normalized on reference gene *UBE2D2*. Values represent mean ± SD and statistical analysis was done using paired ratio t-test. (n ≥ 4; *p < 0.05, **p < 0.01, ***p < 0,001).

The cells regain mesenchymal stromal cell properties, such as multiple differentiation potentials, upon dedifferentiation. The capacity for adipogenic, osteogenic and chondrogenic differentiation was determined in the respective cells to assess the influence of the DNMT inhibitor treatment. Compared to the untreated control, adipogenic and osteogenic differentiation was further improved by 5-aza-dC treatment (Figs 3 and 4, S1 Fig). The staining of the calcified matrix with Alizarin Red as well as lipid vesicles by Oli Red O staining was positive for both populations after differentiation. However, DNMT inhibitor-treated cells displayed a more pronounced lipid vesicle staining and higher expression of FABP4. Furthermore, gene expression of OPN showed increased levels after osteogenic differentiation in cells pretreated with 5-aza-dC (Fig 4).

**Fig 5:**
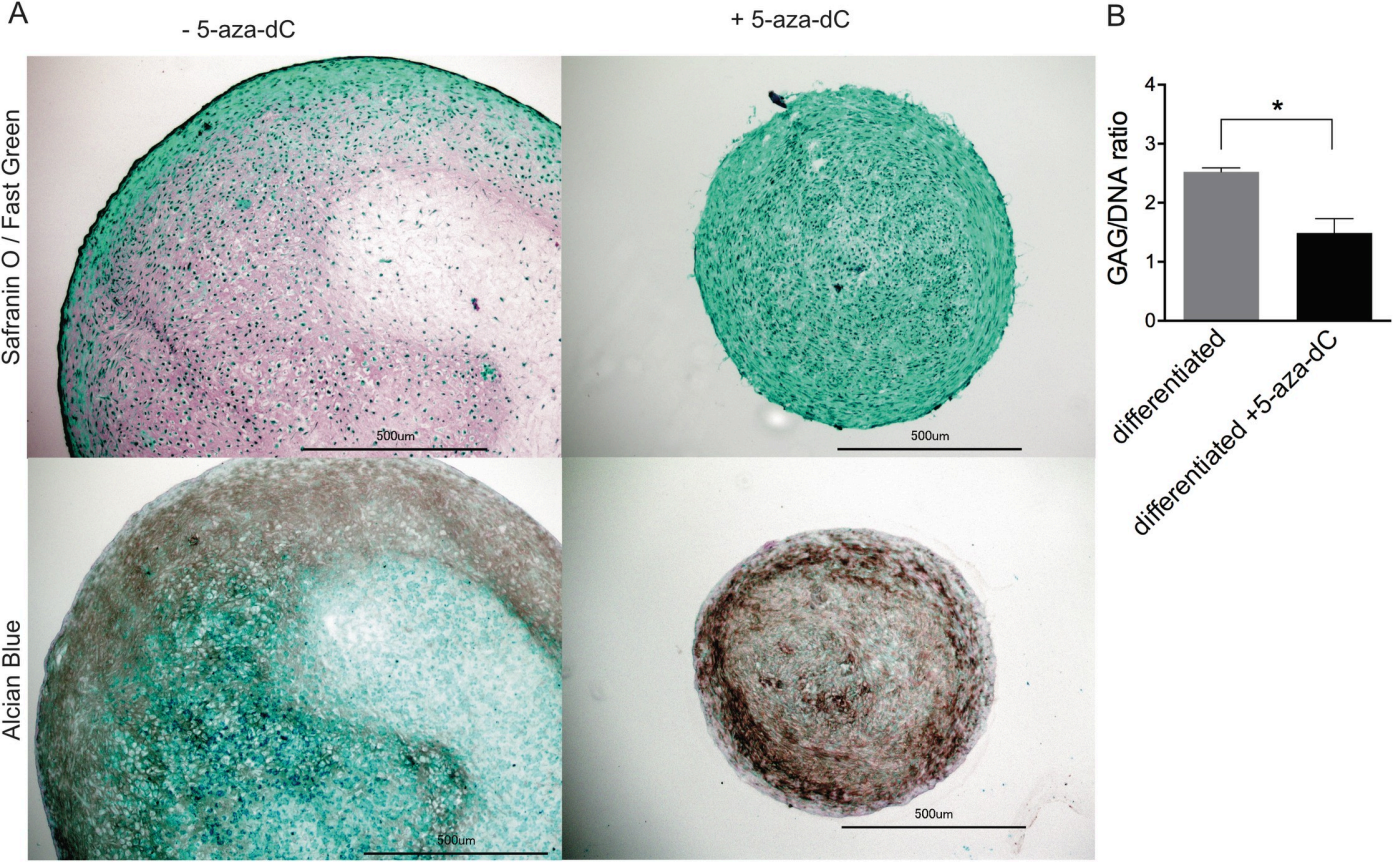
Effect of 5-aza-dC on cartilage matrix formation. After 5-aza-dC treatment, the OA chondrocytes were differentiated towards cartilage for six weeks in three-dimensional culture. (A) Frozen sections were stained for proteoglycans with Alcian Blue and Safranin O / Fast Green. (B) The glycosaminoglycan content was normalized on a DNA amount of the same sample. Values represent mean ± SD and statistical analysis was done using a paired ratio t-test. (n = 3; *p < 0.05).

**Fig 6:**
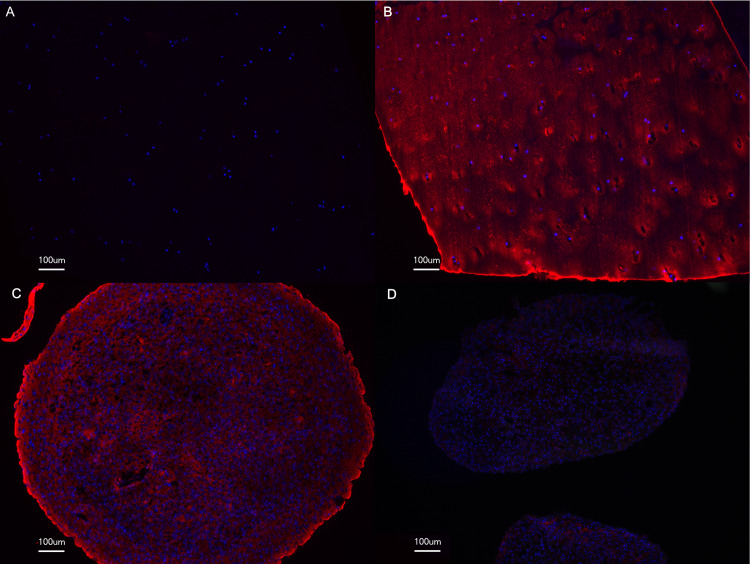
Reduction of collagen type II staining after 5-aza-dC treatment. After 5-aza-dC treatment, the OA chondrocytes were differentiated towards cartilage for six weeks in three-dimensional culture. Immunohistochemistry for collagen type II in cartilage (B), after chondrogenic differentiation with (D) and without (C) 5-aza-dC pre-treatment. Negative control without primary antibody (A).

**Fig 7:**
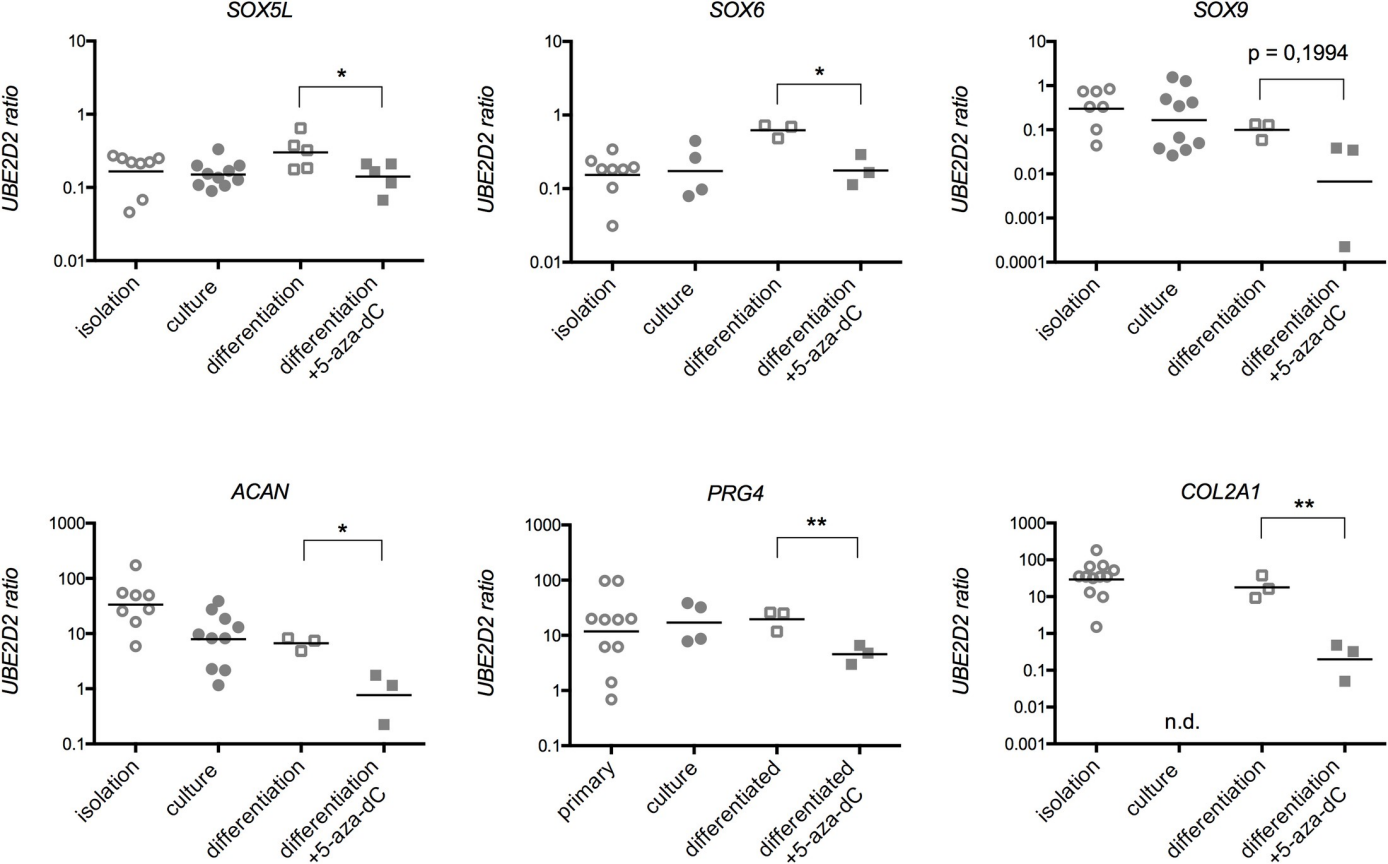
Influence of 5-aza-dC on cartilage marker expression. After 5-aza-dC treatment, the OA chondrocytes were differentiated towards cartilage. After six weeks, the gene expression of specific cartilage markers was measured and normalized on reference gene *UBE2D2*. Values represent mean ± SD and statistical analysis was done using a paired ratio t-test. (n ≥ 4; *p < 0.05, **p < 0.01).

The most striking difference upon 5-aza-dC administration was observed during chondrocyte differentiation. Although no changes in the cartilage marker expression were seen after 5-aza-dC treatment (Fig 1), the chondrogenic differentiation capacity was significantly compromised upon 5-aza-dC treatment (Fig 5). The stimulus of 3D culture and chondrogenic media was not sufficient to induce proper cartilage matrix formation (Fig 5A), and the glycosaminoglycan content was significantly reduced (Fig 5B). The staining of collagen type II was also decreased in the 5-aza-dC treated samples (Fig 6). Furthermore, the coupling of 5-aza-dC expansion with subsequent chondrogenic differentiation reduced the cartilage marker expression (Fig 7). These data strongly indicate that DNMT inhibition impaired a regulator of cartilage formation, whose activity is essential for active chondrogenic differentiation and assumedly dispensable for phenotype maintenance.

**Fig 8:**
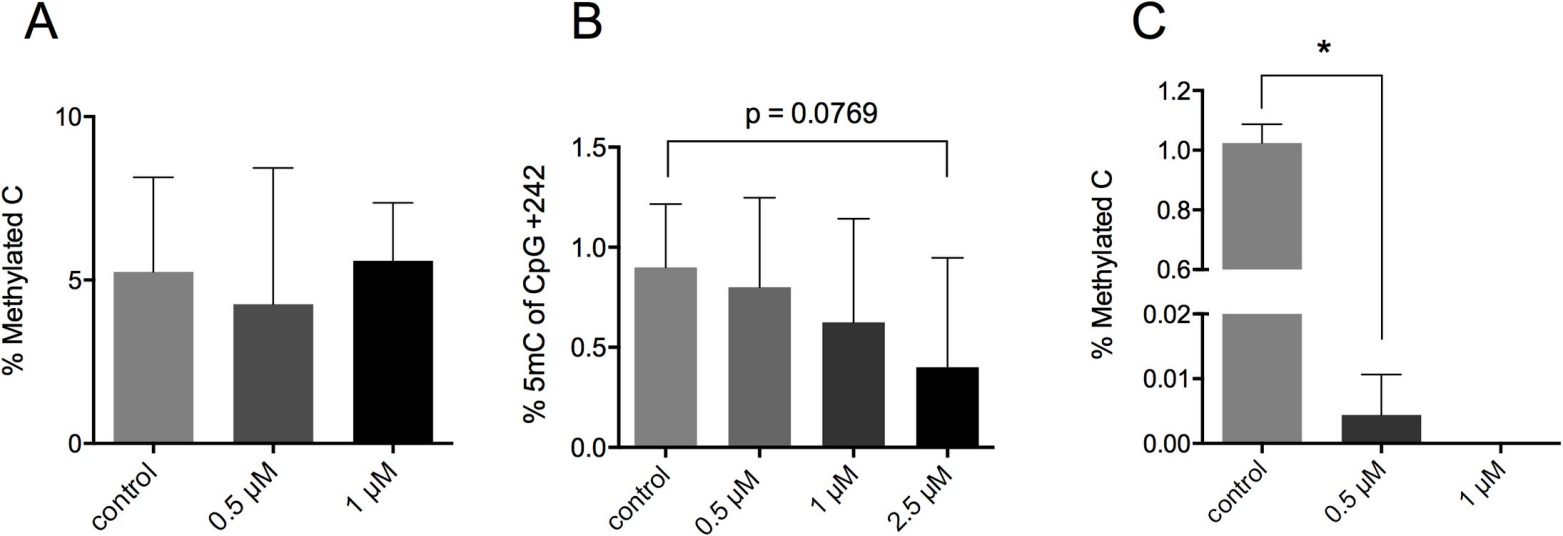
Global DNA methylation level in OA chondrocytes. (A) ELISA of 5-methylcytosine in OA chondrocytes after four weeks of 5-aza-dC treatment. (B) Bisulfite sequencing of CpG at +242 of *PECAM* promoter after four weeks of 5-aza-dC exposure. (C) ELISA of 5-methylcytosine in HEK293T cells after 72 h of 5-aza-dC treatment. Values represent mean ± SD and statistical analysis was done using a student t-test. (n = 4; *p < 0.05).

**Fig 9:**
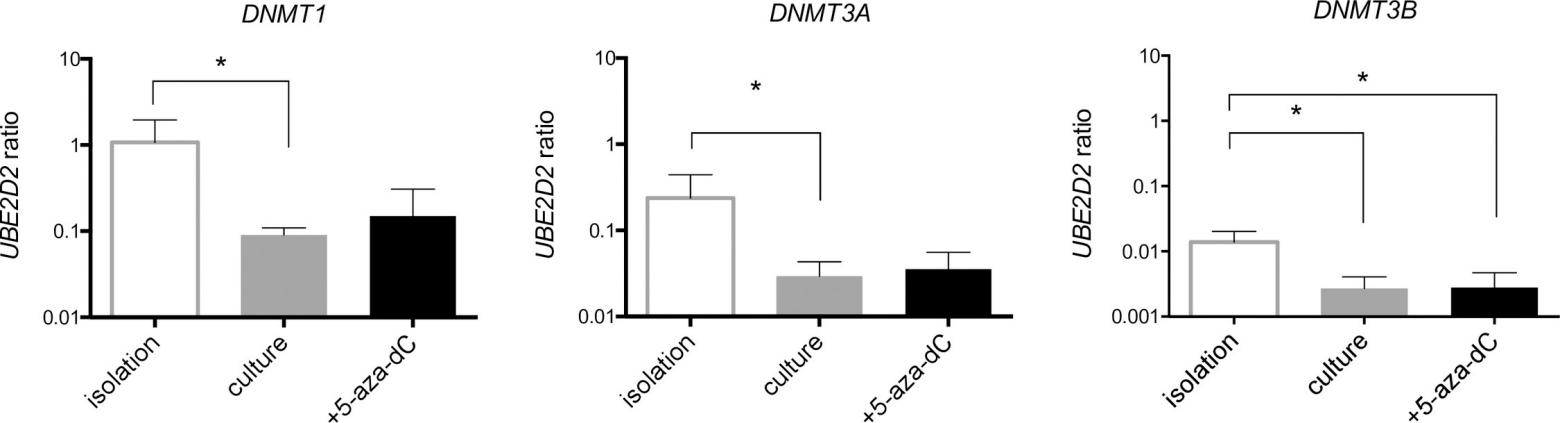
Gene expression of DNA methyltransferases *DNMT1*, *DNMT3A* and *DNMT3B*. Transcription level in isolated OA chondrocytes was compared with dedifferentiated w/o 5-aza-dC treatment (four weeks). Expression was normalized on reference gene *UBE2D2*. Statistical analysis was done using a ratio paired t-test. Values represent mean ± SD (n ≥ 4; *p < 0,05).

The global DNA methylation level was determined comprehensively to assess the effect of DNMT inhibitor treatment on the DNA methylation status. As seen in Fig 8, the long-term treatment of the isolated human OA chondrocytes with 1 μM 5-aza-dC exerted no significant influence on the global methylation level, while the treatment of HEK293T cells with 1 μM 5-aza-dC diminished the DNA methylation after only 72 h. However, the analysis of an individual CpG at site +242 of the PECAM promoter demonstrated, even though not significantly, a tendency of 5-aza-dC as a concentration-dependent DNMT inhibitor by demethylation of the former strongly methylated sequence (Fig 8). The gene expression of DNMTs were analyzed to determine possible influences of cell culture or 5-aza-dC treatment on the gene expression of DNA modifying enzymes. The gene expression of DNMT1, DNMT3a and DNMT3b was downregulated upon expansion *in vitro* with no further impact by additional DNMT inhibitor treatment (Fig 9).

## Discussion

Osteoarthritis is highly prevalent in the elderly population. Implantation of cartilage tissue built from autologous chondrocytes is a treatment which provides pain relief and delays the disease progression [22]. Various biotechnological approaches could improve the cartilage tissue engineering [23]. Several publications report on the dedifferentiation and loss of functionality in healthy chondrocytes upon cell culture [24,25]. In these studies, the focus is mainly on the regenerative potential of chondrocytes. Even though the cartilage we extracted was from even and uninjured areas of the joint, the environmental signaling was, nevertheless, of late-stage OA and should be taken into consideration. The transcriptional differences between healthy and osteoarthritic chondrocytes were described repeatedly [6,24]. In these studies, the loss of cartilage marker expression was reported, as shown in the downregulation of collagen type 2, aggrecan and SOX9, coupled with the upregulation of collagen type 1. Nevertheless, the dedifferentiated chondrocytes showed a high potential for cartilage formation in three-dimensional culture systems. As shown previously, imitating the physiological environment of the tissue without further assistance was sufficient for the dedifferentiated chondrocytes to redifferentiate [26].

In the current study, we demonstrated a maintenance of marker expression in OA chondrocytes after several passages. Our data suggest, that the downregulation had already taken place during disease progression. Interestingly, Lin et al. showed similar gene expression profiles of cultured chondrocytes isolated from healthy and OA joints [27], indicating that both chondrocyte populations were equal after completed dedifferentiation.

For chondrocytes, comparative studies of healthy and OA chondrocytes revealed different DNA methylation patterns [28,29]. In the present study, the treatment of OA chondrocytes with 5-aza-dC led to a decreased cell proliferation even at low doses. Unterberger et al. reported the subjacent mechanisms for the proliferation inhibition observed after DNMT reduction [30]. The dissociation of DNMT1 from the replication fork activates a replication stress checkpoint in an ataxia telangiectasia mutated Rad3-related dependent manner. Through this mechanism, one single incorporated cytosine analog could stall one replication complex until the missing enzyme is replaced.

The increased expression of OPN and FABP4 during osteogenic and adipogenic differentiation after 5-aza-dC treatment are in line with the work of Kim et al. [31], where the chondrogenic, osteogenic and neurogenic differentiation of human bone marrow mesenchymal stem cells was enhanced in the presence of 5-aza-dC. Further studies showed an increase in stemness (expression of markers as *SOX2* and *NANOG*) in cells treated with 5-aza-dC [32,33]. In our study, the 5-aza-dC treatment had the most significant impact on cartilage differentiation. Matrix production was reduced, and marker expression was downregulated. These data are contrary to the work of Duan et al. [5], where the treatment of healthy chondrocytes with 5-aza-C could partially reverse the dedifferentiation of chondrocytes, seen in the increase of the cartilage marker SOX9 and the decrease of collagen type 1 expression restoring the chondrogenic phenotype. Duan et al. isolated chondrocytes from trauma patients, while the chondrocytes in this work were extracted from late-stage OA joints. There, 2 μM 5-aza-C (RNA nucleotide) was used for 24 h, while the cells in this study were treated for four weeks with 1 μM 5-aza-dC (DNA nucleotide). Komashko and Fanham reported the differences between long- and short-term treatment of 5-aza-C on DNA methylation and histone modification patterns [34]. They showed a regulatory effect of the inhibitor mostly on genes which were already unmethylated before treatment and that short-term treatment only slightly reduced the DNA methylation of HEK293 cells. Alvarez-Garcia et al. analyzed the influence of short (48 h) and long-term (four to five weeks) treatment of 5-aza-dC by determining the expression of a few tested markers in chondrocytes, where short-term treatment enhanced the transcription of more genes compared to long-term stimulus [28]. Epigenetic differences between normal and osteoarthritic chondrocytes are described by Alvarez-Garcia et al., where the DNA methylation profiles differ significantly between these two groups [28]. Taken these findings together, the duration of treatment influences transcription by different mechanisms, since the incorporation rate in short-term treatment is very low due to the small number of cell doublings during that time frame.

We demonstrate, the long-term treatment of OA chondrocytes with 5-aza-dC not affect the global DNA methylation levels. Interestingly, the incubation of 5-aza-dC in chondrocytes in the work of Hashimoto et al. leads to a decrease of DNA methylation of specific CpGs within the IL1b promoter, while global DNA demethylation was not shown [35]. In other studies, the methylome analysis by next-generation sequencing illustrates the differences in DNA methylations of chondrocytes at different passages as well as 5-aza-dC treated versus untreated cells [5]. Their findings indicate a directed change in DNA methylation, since the regions which were hypomethylated by 5-aza-dC most prominently were hypermethylated upon artificial cell culture. Furthermore, the treatment of 5-aza-dC in primary healthy chondrocytes indeed demethylate DNA but preferably of distinct and possibly cartilage-specific regions. Our data and the study by Hagemann et al. indicate, that the DNA demethylation was found to be nonrandom and reproducible [36]. They reported that specific CpGs within CpG islands become re-methylated upon treatment with 5-aza-dC and, furthermore, they identified sequences which were never affected by treatment at all [36]. The inhibiting effect of 5-aza-dC on cartilage marker expression during chondrogenesis is the focus of further investigations. Whole genome methylation analysis is required to identify the key player of the changes observed regarding the duration of treatment.

Without fully exploring the mechanisms behind the cartilage marker maintenance in OA chondrocytes, their beneficial properties represent a helpful tool for *in vitro* applications. The present study provides evidence that late-stage OA chondrocytes have a high cartilage formation capacity, and our data are in line with multiple publications which report that OA chondrocytes are a source of osteochondroprogenitors and have equal capacity to healthy chondrocytes [37–40].

## Conclusion

Chondrocytes isolated from late-stage OA do not show the same changes as healthy chondrocytes during dedifferentiation upon cell culture. They can differentiate towards adipogenic, osteogenic or chondrogenic lineages. The adipogenic and osteogenic differentiation was further enhanced under long-term treatment with DNMT inhibitor 5-aza-dC, while the proliferation of cells slowed down. On the other hand, the chondrogenic differentiation was inhibited. Although the DNMT inhibitor was added every second day for four weeks during the expansion of cells, no global DNA demethylation could be observed, indicating an additional independent mechanism responsible for the effects observed.

## Supporting information

S1 Table qPCR primer. Nucleotide sequence of the qPCR primers used in this study.
(DOCX)

S1 Fig Adipogenic and osteogenic marker expression. After 5-aza-dC treatment, the OA chondrocytes were differentiated towards osteoblasts and adipocytes, respectively. The marker expression of *PPARg*, *LPL*, *RUNX2 and OC* was measured and normalized on reference gene *UBE2D2*.
(TIF)

## References

[1] Ikegami K, Ohgane J, Tanaka S, Yagi S, Shiota K. Interplay between DNA methylation, histone modification and chromatin remodeling in stem cells and during development. Int J Dev Biol. 2009;53: 203–214. 10.1387/ijdb.082741ki 19412882

[2] Avgustinova A, Benitah SA. Epigenetic control of adult stem cell function. Nat Rev Mol Cell Biol. 2016;17: 643–658. 10.1038/nrm.2016.76 27405257

[3] Yamada Y, Haga H, Yamada Y. Concise Review: Dedifferentiation Meets Cancer Development: Proof of Concept for Epigenetic Cancer. Stem Cells Transl Med. 2014;3: 1182–1187. 10.5966/sctm.2014-0090 25122691 PMC4181402

[4] Kohli RM, Zhang Y. TET enzymes, TDG and the dynamics of DNA demethylation. Nature. 2013;502: 472 10.1038/nature12750 24153300 PMC4046508

[5] Duan L, Liang Y, Ma B, Wang D, Liu W, Huang J, et al DNA Methylation Profiling in Chondrocyte Dedifferentiation In Vitro. J Cell Physiol. 2017;232: 1708–1716. 10.1002/jcp.25486 27404036

[6] Ma B, Leijten JCH, Wu L, Kip M, van Blitterswijk CA, Post JN, et al Gene expression profiling of dedifferentiated human articular chondrocytes in monolayer culture. Osteoarthr Cartil. 2013;21: 599–603. 10.1016/j.joca.2013.01.014 23376013

[7] Barter MJ, Bui C, Young DA. Epigenetic mechanisms in cartilage and osteoarthritis: DNA methylation, histone modifications and microRNAs. Osteoarthr Cartil. 2012;20: 339–349. 10.1016/j.joca.2011.12.012 22281264

[8] Im G-I, Choi Y-J. Epigenetics in osteoarthritis and its implication for future therapeutics. Expert Opin Biol Ther. 2013;13: 713–721. 10.1517/14712598.2013.764410 23410522

[9] Pohlig F, Guell F, Lenze U, Lenze FW, Mühlhofer HML, Schauwecker J, et al Hyaluronic Acid Suppresses the Expression of Metalloproteinases in Osteoarthritic Cartilage Stimulated Simultaneously by Interleukin 1β and Mechanical Load. PLoS One. 2016;11: e0150020 10.1371/journal.pone.0150020 26934732 PMC4774918

[10] Goldring SR, Goldring MB. Changes in the osteochondral unit during osteoarthritis: structure, function and cartilage-bone crosstalk. Nat Rev Rheumatol. 2016;12: 632–644. 10.1038/nrrheum.2016.148 27652499

[11] Doran PM. Cartilage Tissue Engineering: What Have We Learned in Practice? Methods Mol Biol. 2015;1340: 3–21. 10.1007/978-1-4939-2938-2_1 26445827

[12] Ahmed TAE, Hincke MT. Mesenchymal stem cell—Based tissue engineering strategies for repair of articular cartilage. Histology and Histopathology. 2014 pp. 669–689. 10.14670/HH-29.669 24452855

[13] Long F, Ornitz DM. Development of the endochondral skeleton. Cold Spring Harb Perspect Biol. 2013;5: a008334 10.1101/cshperspect.a008334 23284041 PMC3579395

[14] Decker RS, Koyama E, Pacifici M. Genesis and morphogenesis of limb synovial joints and articular cartilage. Matrix Biol. 2014;39: 5–10. 10.1016/j.matbio.2014.08.006 25172830 PMC4198612

[15] Goldring MB, Goldring SR. Articular cartilage and subchondral bone in the pathogenesis of osteoarthritis. Ann N Y Acad Sci. 2010;1192: 230–237. 10.1111/j.1749-6632.2009.05240.x 20392241

[16] Christman JK. 5-Azacytidine and 5-aza-2′-deoxycytidine as inhibitors of DNA methylation: Mechanistic studies and their implications for cancer therapy. Oncogene. 2002;21: 5483–5495. 10.1038/sj.onc.1205699 12154409

[17] Stresemann C, Lyko F. Modes of action of the DNA methyltransferase inhibitors azacytidine and decitabine. Int J Cancer. 2008;123: 8–13. 10.1002/ijc.23607 18425818

[18] Cameron EE, Bachman KE, Myöhänen S, Herman JG, Baylin SB. Synergy of demethylation and histone deacetylase inhibition in the re-expression of genes silenced in cancer. Nat Genet. 1999;21: 103–107. 10.1038/5047 9916800

[19] Koressaar T, Remm M. Enhancements and modifications of primer design program Primer3. Bioinformatics. 2007;23: 1289–1291. 10.1093/bioinformatics/btm091 17379693

[20] Untergasser A, Cutcutache I, Koressaar T, Ye J, Faircloth BC, Remm M, et al Primer3—new capabilities and interfaces. Nucleic Acids Res. 2012;40 10.1093/nar/gks596 22730293 PMC3424584

[21] Momparler RL. Pharmacology of 5-aza-2′-deoxycytidine (decitabine). Semin Hematol. 2005;42: S9–S16. 10.1053/j.seminhematol.2005.05.002 16015507

[22] Zhang W, Ouyang H, Dass CR, Xu J. Current research on pharmacologic and regenerative therapies for osteoarthritis. Bone Res. 2016;4: 15040 10.1038/boneres.2015.40 26962464 PMC4772471

[23] Stellavato A, Tirino V, de Novellis F, Della Vecchia A, Cinquegrani F, De Rosa M, et al Biotechnological Chondroitin a Novel Glycosamminoglycan With Remarkable Biological Function on Human Primary Chondrocytes. J Cell Biochem. 2016;117: 2158–2169. 10.1002/jcb.25556 27018169 PMC5084766

[24] Duan L, Ma B, Liang Y, Chen J, Zhu W, Li M, et al Cytokine networking of chondrocyte dedifferentiation in vitro and its implications for cell-based cartilage therapy. Am J Transl Res. 2015;7: 194–208. Available: https://www.ncbi.nlm.nih.gov/pubmed/25901191 25901191 PMC4399086

[25] Demoor M, Ollitrault D, Gomez-Leduc T, Bouyoucef M, Hervieu M, Fabre H, et al Cartilage tissue engineering: Molecular control of chondrocyte differentiation for proper cartilage matrix reconstruction. Biochim Biophys Acta—Gen Subj. 2014;1840: 2414–2440. 10.1016/j.bbagen.2014.02.030 24608030

[26] Rosowski M, Falb M, Tschirschmann M, Lauster R. Initiation of mesenchymal condensation in alginate hollow spheres—a useful model for understanding cartilage repair? Artif Organs. 2006;30: 775–784. 10.1111/j.1525-1594.2006.00300.x 17026577

[27] Lin Z, Fitzgerald JB, Xu J, Willers C, Wood D, Grodzinsky AJ, et al Gene expression profiles of human chondrocytes during passaged monolayer cultivation. J Orthop Res. 2008 10.1002/jor.20523 18404652

[28] Alvarez-Garcia O, Fisch KM, Wineinger NE, Akagi R, Saito M, Sasho T, et al Increased DNA Methylation and Reduced Expression of Transcription Factors in Human Osteoarthritis Cartilage. Arthritis Rheumatol. 2016;68: 1876–1886. 10.1002/art.39643 26881698 PMC4963260

[29] Bonin CA, Lewallen EA, Baheti S, Bradley EW, Stuart MJ, Berry DJ, et al Identification of differentially methylated regions in new genes associated with knee osteoarthritis. Gene. 2016;576: 312–318. 10.1016/j.gene.2015.10.037 26484395 PMC4679536

[30] Unterberger A, Andrews SD, Weaver ICG, Szyf M. DNA methyltransferase 1 knockdown activates a replication stress checkpoint. Mol Cell Biol. 2006;26: 7575–86. 10.1128/MCB.01887-05 17015478 PMC1636877

[31] Kim HJ, Kwon Y-RR, Bae Y-JJ, Kim Y-JJ. Enhancement of human mesenchymal stem cell differentiation by combination treatment with 5-azacytidine and trichostatin A. Biotechnol Lett. 2016;38: 167–174. 10.1007/s10529-015-1949-3 26341652

[32] Liang X, Xu C, Wang W, Li X. The DNMT1/miR-34a Axis Is Involved in the Stemness of Human Osteosarcoma Cells and Derived Stem-Like Cells. Stem Cells Int. 2019;2019 10.1155/2019/7028901 31781245 PMC6875320

[33] La Noce M, Paino F, Mele L, Papaccio G, Regad T, Lombardi A, et al HDAC2 depletion promotes osteosarcoma’s stemness both in vitro and in vivo: a study on a putative new target for CSCs directed therapy. J Exp Clin Cancer Res. 2018;37: 296 10.1186/s13046-018-0978-x 30509303 PMC6276256

[34] Komashko VM, Farnham PJ. 5-azacytidine treatment reorganizes genomic histone modification patterns. Epigenetics. 2010;5: 229–240. 10.4161/epi.5.3.11409 20305384

[35] Hashimoto K, Oreffo ROC, Gibson MB, Goldring MB, Roach HI. DNA demethylation at specific CpG sites in the IL1B promoter in response to inflammatory cytokines in human articular chondrocytes. Arthritis Rheum. 2009;60: 3303–3313. 10.1002/art.24882 19877066 PMC2788707

[36] Hagemann S, Heil O, Lyko F, Brueckner B. Azacytidine and decitabine induce gene-specific and non-random DNA demethylation in human cancer cell lines. PLoS One. 2011;6 10.1371/journal.pone.0017388 21408221 PMC3049766

[37] Jiang Y, Tuan RS. Origin and function of cartilage stem/progenitor cells in osteoarthritis. Nat Rev Rheumatol. 2015;11: 206–212. 10.1038/nrrheum.2014.200 25536487 PMC5413931

[38] Khan IM, Williams R, Archer CW. One flew over the progenitor’s nest: migratory cells find a home in osteoarthritic cartilage. Cell Stem Cell. 2009;4: 282–284. 10.1016/j.stem.2009.03.007 19341617

[39] Koelling S, Kruegel J, Irmer M, Path JR, Sadowski B, Miro X, et al Migratory chondrogenic progenitor cells from repair tissue during the later stages of human osteoarthritis. Cell Stem Cell. 2009;4: 324–335. 10.1016/j.stem.2009.01.015 19341622

[40] Oda T, Sakai T, Hiraiwa H, Hamada T, Ono Y, Nakashima M, et al Osteoarthritis-derived chondrocytes are a potential source of multipotent progenitor cells for cartilage tissue engineering. Biochem Biophys Res Commun. 2016;479: 469–475. 10.1016/j.bbrc.2016.09.085 27644879

