# Supplementary material for: Effects of 5-aza-2´-deoxycytidine on primary human chondrocytes from osteoarthritic patients
Source: PLoS One. 2020 Jun 23;15(6):e0234641. doi: 10.1371/journal.pone.0234641 (PMC7310740; doi:10.1371/journal.pone.0234641)
Supplement: S1 Table — Nucleotide sequence of the qPCR primers used in this study. (DOCX) [file pone.0234641.s001.docx]

| Identifier | target | primer up | primer down |
| --- | --- | --- | --- |
| NM_001135 | *ACAN* | GGGATGGTGGATGTCAGTTGG | CTCCTGCCTCTTGGGCTGTT |
| NM_000493 | *COL10A1* | CCACTACCCAACAACAAGACACA | GGCAACCCTGGCTCTCCTT |
| NM_001844 | *COL2A1* | ACTCAAGTCCCTCAACAACCAG | CTGCTCCACCAGTTCTTCTTG |
| NM_000095 | *COMP* | GACAAGAAGTCCTATCGTTGGTTCC | TCATAGTCCTCTGGGATGGTGTC |
| NM_004230 | *DNMT1* | GGTGGTGGATGACAAGAAGTTTG | TGAGGATGGGCTGGTACTGTG |
| NM_175629 | *DNMT3A* | TCCAACCCTGTGATGATTGATG | CTTTGCTGAACTTGGCTATCCTG |
| NM_006892 | *DNMT3B* | CCGGTGTTTCTGTGTGGAGTG | CTTCATATTCAAGCCCCGTGTC |
| NM_001442 | *FABP4* | GCAGAAATGGGATGGAAAATCA | CGTCCCTTGGCTTATGCTCTC |
| NM_000582 | *OPN* | CACTGATTTTCCCACGGACCT | CCATTCAACTCCTCGCTTTCC |
| NM_005807 | *PRG4* | CCATGCTTTCCGATGAGACC | CAATGGGGGAAGGAATACCC |
| NM_001015051 | *RUNX2* | TCACAAATCCTCCCCAAGTAGC | GGCGGGACACCTACTCTCATAC |
| NM_152989 | *SOX5 isoform b* | CGAGCCACCAAAACCCATC | TCAGCAAGAGGAAAGCCCAGTAG |
| NM_006940 | *SOX5 isoform a* | TTGACAGGTTCAGTTGGAGACG | GAGTGAGGCTTGTTGGGAAAAC |
| NM_017508 | *SOX6* | TAAATACAAACCCCGACCGAAAC | GATAGCACCAGGATACACAACACCT |
| NM_000346 | *SOX9* | GCCAGGTGCTCAAAGGCTAC | CGCTTCTCGCTCTCGTTCA |
| NM_003254 | *TIMP1* | ACTTCCACAGGTCCCACAACC | TTTGCAGGGGATGGATAAACAG |
| NM_003255 | *TIMP2* | CGTCTCCCGTCTTTGGTTCTC | CACCCGGCTCTTCTTAACCTG |
| NM_181838 | *UBE2D2* | TCAGGCACTAAAGGATCATCTGG | TCTTGACAATTCATTTCCCAACAG |
