# Supplementary figures and images for: Effects of 5-aza-2´-deoxycytidine on primary human chondrocytes from osteoarthritic patients
Source: PLoS One. 2020 Jun 23;15(6):e0234641. doi: 10.1371/journal.pone.0234641 (PMC7310740; doi:10.1371/journal.pone.0234641)

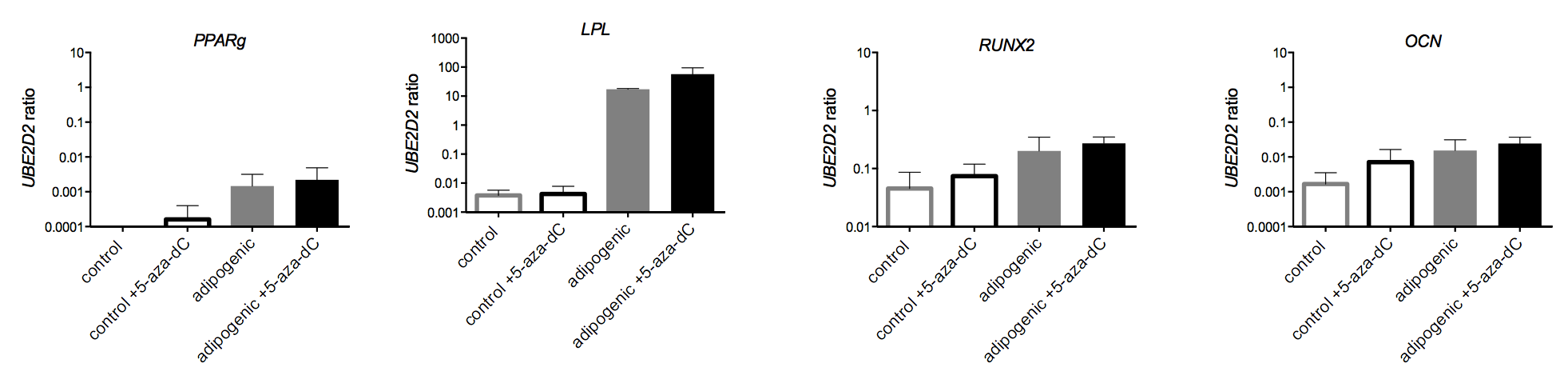

Supplement: S1 Fig — After 5-aza-dC treatment, the OA chondrocytes were differentiated towards osteoblasts and adipocytes, respectively. The marker expression of PPARg, LPL, RUNX2 and OC was measured and normalized on reference gene UBE2D2. (TIF) [file pone.0234641.s002.tif]
